# Personality traits and their correlation with behavior in the bonnet macaque (*Macaca radiata*) in southern India

**DOI:** 10.1038/s41598-024-82283-0

**Published:** 2024-12-28

**Authors:** Małgorzata E Arlet, Olga Sakson-Obada, Marzena Zakrzewska, Mewa Singh

**Affiliations:** 1 Institute of Human Biology and Evolution, Faculty of Biology, Adam Mickiewicz University in Poznań, Poznań, Poland; 2 Faculty of Psychology and Cognitive Science, Adam Mickiewicz University in Poznań, Poznań, Poland; 3 Department of Studies in Psychology, University of Mysore, Mysore, India

**Keywords:** Personality traits, Behavior, Sex, Age, Dominance rank, Psychology, Zoology

## Abstract

The purpose of our research was to identify the primary personality traits of bonnet macaques using an observer-based questionnaire and to assess the accuracy of this method in predicting behavior. Additionally, we examined the relationship between personality traits and variables such as sex, age, and rank. We rated 25 males and 38 females on 50 personality questionnaire items using a 7-point scale, with reliable ratings and good agreement among raters (the lowest of the calculated ICC (3:k) index ≥ 0.62). Behavioral observations of 38 females over two-years were analyzed. Exploratory factor analysis revealed five traits: Friendliness, Aggression, Playfulness, Fearfulness, and Opportunism. Every trait correlated with a certain number of observed behaviors. Our results suggest that the questionnaire-based method is sufficiently valid as it confirmed high behavioral convergence with Fearfulness, Playfulness and Aggressiveness. Further studies are needed to understand the functional value of behaviors associated with Friendliness and Opportunism. Our study confirmed a tolerant social style of bonnet macaques, characterized by similar Aggression levels in both males and females, and lack of difference in Aggression level between subadult and adult males. Moreover, we identified a species-characteristic trait Opportunism which, unlike in other studied macaques, did not involve aggression-related characteristics.

**Supplementary Information:**

The online version contains supplementary material available at 10.1038/s41598-024-82283-0.

## Introduction

The interest in quantifying consistent personality in nonhuman primate (hereinafter, primates) behavior has grown rapidly^1–4^. Personality variation is usually subsumed by a limited number of traits/dimensions, which, along with characteristics of the specific situation, contribute to the individual expression in that situation^5,6^. Personality traits are defined as a general pattern of decision making, thinking, feeling, and acting which is genetically passed on, and is dependent on biological changes associated with maturation^7^. Personality differences are thought to be beneficial within populations, as they promote task differentiation, including roles like exploration and vigilance, which can enhance overall adaptability through frequency-dependent selection^8–11^. Such adaptive individual differences emerged via natural selection and reflect the challenges that the animal has faced on its evolutionary path^4,12,13^. Therefore, despite the emphasis on the genetic basis of traits, interaction with the environment is crucial for their expression^4,14^. Environmental factors like parental care style, social status, dispersal, and birth order modulate innate predispositions^4,15^.

The studies of personality in primates are based on the assessments of specific behaviors (e.g. behavioral coding) or characteristics in the form of ratings^2^. Observing different primate species, their habitats (wild or captive), the varying protocols, and species-specific dimensions has led researchers to analyze a different number of traits in each study, even within the same species (cf. the number of traits distinguished in rhesus macaques: three in the study by Stevenson-Hinde & Zunz^16^, van Borell^17^; four in the study by Capitanio^5^; five in the study by Weiss et al.^18^; six in the study by Brent et al.^19^). Comparison of questionnaire-based studies on macaques suggests that the more questionnaire items included in the factor analysis, the more personality traits were identified. Thus, it remains unclear whether the number of detected personality traits is species-specific or dependent on their environment, or the methodology used. Despite that variation, studies of the macaque species most often identify traits such as Sociability/Friendliness, Fearfulness, Activity/ Exploration/Playfulness, and Aggression/Dominance^2,20^.

Personality traits are associated with age, sex and social position^21,22^. Some of these differences seem to be consistent among the species^23^. The significance of age in relation to personality changes (i.e., personality stability) considered both the comparison of the average intensity of a trait across different age groups (mean-level stability) and the results of longitudinal studies of individual subjects (rank-order stability^14,24^). The results of the rank-order stability studies suggest that, similar to humans^24^, personality tends to consolidate during adulthood not only in macaques, but in primates in general^16,17,25^. Longitudinal studies among rhesus macaques (*Macaca mulatta*) have proven that traits such as Aggression/Confidence and Sociability consolidate in adulthood, while Fearfulness is relatively stable^16,21^. Research on mean-level change in personality in primates suggests that trends for some traits are universal, while others are species-specific and influenced by sex. It has been demonstrated that primates show a consistent pattern of decline in traits related to exploration with age, whereas the direction of change in Agreeableness was not consistent across species (for review, see^14^). Research on mean-order stability in captive pigtailed macaques (*M. nemestrina*) has demonstrated that with aging, individuals become less Cautious and more Aggressive^25^. The increase in Aggressiveness/Dominance among older male rhesus macaques has been confirmed in another study^16^, which also revealed an interaction between sex and age in influencing personality changes. Specifically, adult males were more confident and, at the same time, more solitary than younger males and adult females (mean-level change)^16^. Due to generally lower Aggressiveness in females compared to males, age is unlikely to affect the intensity of this trait in females. Findings from studies on free-ranging female rhesus macaques^19^ suggested that age did not significantly impact dominance and aggression. These results emphasize the importance of examining the interaction between sex and age when assessing mean-level changes.

Similarly to humans, research on sex differences in primates shows that Agreeableness and Sociability were higher in females, while Dominance, Activity and Assertiveness were higher in males^26,27^. However, it should be noted that the relationship between personality and sex is not always clear, since it can be modulated by the age of the individual, the level of tolerance and stability of the social hierarchy, the heredity of status and the mobility of each sex^26,28,29^. The contribution of these modulators explains the varied findings on the relationship between sex and personality in primates, as for example, studies of vervet monkeys (*Chlorocebus pygerythrus*) showed that age and sex, were linked to personality traits in stable groups^29^.

Becoming a high-ranking individual relates to physiological and psychological changes^30^, which can lead to modification in behavior. When investigating the relation between personality and social rank in rhesus macaques^16^and pig-tailed macaques^31^, researchers rated dominant individuals as Confident and Effective. In contrast, observers characterized subordinate rhesus macaques as Insecure, Dependent, and less Popular. High-ranking females in the same species exhibited greater Popularity (and Confidence) during the reproductive phase^16^.

Most of the studies on personality in primates are from the captive populations, though the natural environment of a species facilitates full expression of their behavioral repertoire. Studying animals in natural habitats allows consideration of factors such as migration, intergroup encounters and predators, which are absent in captivity but essential for accurate personality assessment. Here, we report the study of a free ranging population of bonnet macaques (*M. radiata*) in southern India. The purpose of our research was to identify the primary personality traits of bonnet macaques through observer questionnaire assessments and to explore the accuracy of this method in predicting behavior. Additionally, we examined the relationship between personality traits and the sex, age, and rank of individuals.

We expect that traits such as Fearfulness, Friendliness/Sociability, Exploration/Playfulness and Aggressiveness/Dominance that were observed in other macaque species^5,17,19,32^, will be present in our study population. Bonnet macaques stand apart from other macaque species by possessing a unique mix of social traits. Their social style falls somewhere between “despotic” and “tolerant” - or Grade 3 - in Thierry’s classification system, showing both dominating and egalitarian behavior^33–35^. Bonnet macaques typically live in multi-male, multi-female groups, in which females are philopatric and most males disperse when they reach sexual maturity^36^. We predict that since females in this species form matrilineal bonds^37^, their Sociability will be higher, in contrast to dispersing males. In bonnet macaques, male-male relationships play an integral part in forming group social dynamics. While other macaque species exhibit rigid dominance hierarchies, bonnet macaque males form strong affiliative bonds between themselves^38,39^. Interestingly, even immigrant males belonging to non-natal groups demonstrated significantly more grooming behavior and spatial associations compared with their natal counterparts, likely creating strong social bonds within their new troop^38^. Therefore, we expect that high tolerance among males will lead to a low level of aggression, comparable to that in females. We expect that subadult individuals will be more playful than adults. Moreover, we predicted that higher rank would be related to higher Aggressiveness/Dominance, and lower rank, to higher Fearfulness.

## Results

All statistical analyses were performed using the IBM SPSS Statistics package (version 29.0.2.0).

### Interrater agreement

Inter-rater reliability was measured with the ICC (3:k) index (two way mixed effects, consistency, multiple raters). Individuals were observed by a varying number of raters (ranging from two to six). The ICC indexes ranged from 0.62 to 0.96 and were acceptable, leading to use of all available data. Detailed information is included in the Appendix (Table A1). In this table, ICC (3:k) ratios are accompanied by ICC (3:1) estimates.

It is usually assumed that a sufficient level of consistency for ICC (3:k) equals 0.40^40^. However, Weiss^41^ pointed out the proposal to accept all values of ICC indicators that are greater than zero when a factor analysis is planned for the obtained estimates. In our study none of the 95% Confidence Intervals for the ICC population values contained zero and the smallest ICC (3:k) value was 0.62. Analysis of the averaged ICC indices showed that, regardless of the number of observers, the consistency of the raters is sufficient. Therefore, the final adjective ratings were constructed by taking into account the average value for all raters (from 2 to 6) observing a particular monkey.

### Factor analysis

Fifty trait ratings were first submitted to measures of sampling adequacy. The overall Kaiser-Meyer-Olkin (KMO) index for all traits was 0.64 and was acceptable^42,43^. KMO indexes for individual traits ranged from 0.44 to 0.89 (Table A1 in the Appendix) and were also acceptable. Bartlett’s test of sphericity was significant (χ^2^_(1225)_ = 3446.513; *p* < 0.001).

Fifty ratings on questionnaires were subjected to exploratory factor analysis. The analyses were conducted on 63 monkeys, including: 32 (50.8%) adult females, 18 (28.6%) adult males, 6 (9.5%) subadult females and 7 (11.1%) subadult males. The sampling size was adequate in the terms of the group structure of bonnet macaque. The components were extracted with a principal components method. This analysis revealed five factors accounting for 70% of the total variance. The number of factors was determined based on the analysis of the eigenvalues of the successive components (the scree test). As reported in Table 1, the next factors explain about 30, 21, 8, 6 and 5% of the variance in the output variables. Extracted factors were next rotated. Orthogonal and oblique (oblimin) rotation produced very similar patterns. It was decided to use an oblique transformation because the orthogonal solution imposes a lack of correlation between the extracted factors. If, indeed, the extracted components are orthogonal, then using oblique rotation, the extra-diagonal elements of the correlation matrix between the factors will be close to zero. The oblique solution (after oblimin rotation) of the factors is shown in Table 1 and the correlation matrix between the extracted factors is included in the Appendix (Table A2).

**Table 1:** Factor structure of 50 traits ratings (adjectives) after oblimin rotation.

Item	Fearfulness	Friendliness	Playfulness	Aggressiveness	Opportunism	Communalities
Nervous	**− 0.946**	− 0.012	− 0.075	0.157	0.027	0.855
Tense	**− 0.900**	0.004	− 0.051	0.261	− 0.009	0.780
Timid	**− 0.858**	− 0.127	0.068	− 0.114	− 0.134	0.785
Fearful	**− 0.805**	0.178	0.199	− 0.038	− 0.171	0.824
Apprehensive	**− 0.777**	− 0.008	− 0.118	0.025	0.063	0.619
Insecure	**− 0.741**	0.129	− 0.183	0.098	− 0.152	0.730
Submissive	**− 0.736**	− 0.082	0.333	− 0.389	− 0.039	0.823
Solitary	**− 0.660**	0.025	− 0.052	− 0.098	− 0.181	0.572
Cautious	**− 0.572**	− 0.088	− 0.125	− 0.375	0.304	0.608
Nurturant	− 0.066	**− 0.819**	− 0.136	− 0.026	0.233	0.677
Understanding	− 0.057	**− 0.824**	− 0.142	− 0.157	0.014	0.731
Protective	0.174	**− 0.740**	0.120	0.037	− 0.039	0.691
Warm	0.267	**− 0.728**	0.106	− 0.188	0.055	0.807
Unemotional	0.133	**0.633**	− 0.286	− 0.027	− 0.414	0.649
Gentle	0.011	**− 0.710**	0.051	− 0.266	− 0.326	0.782
Tolerant	− 0.092	**− 0.684**	0.049	− 0.373	− 0.013	0.676
Sensitive	− 0.179	**− 0.673**	− 0.081	− 0.067	− 0.126	0.470
Affiliative	0.257	**− 0.627**	0.224	0.223	0.124	0.736
Popular	0.435	**− 0.496**	0.177	0.240	− 0.159	0.714
Sociable	0.281	**− 0.491**	0.241	0.173	− 0.119	0.537
Playful	− 0.086	− 0.084	**0.888**	0.075	− 0.244	0.793
Active	− 0.070	− 0.244	**0.697**	0.075	0.315	0.745
Slow	− 0.351	− 0.189	**− 0.618**	0.294	− 0.358	714
Curious	0.421	− 0.182	**0.571**	0.183	− 0.089	0.738
Independent	0.186	0.146	**0.397**	0.083	0.199	0.321
Irritable	− 0.095	0.136	− 0.215	**0.842**	− 0.008	0.738
Aggressive	0.097	0.161	− 0.138	**0.789**	0.145	0.759
Bullying	0.102	0.066	− 0.129	**0.789**	0.037	0.678
Manipulative	− 0.142	0.168	0.063	**0.762**	− 0.107	0.638
Feisty	0.046	0.009	0.253	**0.723**	0.183	0.760
Jealous	− 0.114	0.095	0.066	**0.709**	0.203	0.613
Unpredictable	− 0.250	0.194	0.149	**0.680**	0.206	0.678
Defiant	− 0.038	0.030	0.222	**0.672**	− 0.132	0.528
Impulsive	0.001	0.280	0.451	**0.660**	0.054	0.878
Direct	0.366	− 0.229	− 0.167	**0.689**	− 0.058	0.743
Reckless	0.052	0.243	0.431	**0.641**	− 0.052	0.780
Effective	0.404	− 0.203	− 0.171	**0.668**	− 0.120	0.745
Persistent	0.087	− 0.353	0.283	**0.669**	0.071	0.743
Stingy	0.304	0.140	− 0.294	**0.601**	0.173	0.627
Strong	0.357	− 0.239	0.003	**0.625**	− 0.187	0.677
Excitable	− 0.210	0.168	0.369	**0.551**	0.032	0.554
Bold	0.428	− 0.197	0.227	**0.514**	0.093	0.777
Vigilant	− 0.016	0.096	− 0.148	0.108	**0.762**	0.609
Lazy	− 0.172	0.242	− 0.164	0.130	**− 0.731**	0.738
Depressed	− 0.391	− 0.017	− 0.185	0.140	**− 0.592**	0.614
Opportunistic	0.061	− 0.239	0.271	0.407	**0.530**	0.736
Equable	0.212	− 0.435	− 0.016	− 0.197	**− 0.519**	0.625
Intelligent	0.256	− 0.457	− 0.136	0.110	**0.484**	0.594
Calm*	0.228	− 0.451	− 0.074	− 0.481	− 0.331	0.730
Confident*	0.505	− 0.411	− 0.030	0.455	0.031	0.811
Variance explained	30%	21%	8%	6%	5%	
Cronbach’s alpha	0.93	0.91	0.79	0.95	0.76	
SEM	0.24	0.22	0.44	0.21	0.30	

*Adjectives not used in the process of creating personality scales as they defined (factor loading > 0.40) two factors simultaneously.

In the correlation matrix between the factors (Table A2), we noted the highest values for factor one. With factor two and with factor four it had the most common variance (about 10 and 6%, respectively). The weakest correlations characterized factor two. With factor three and with factor four it had 0.7 and 0.1% common variance, respectively. The remaining pairs of extracted factors had approximately 1 to 3% of the variance in common. The average correlation coefficient of the first factor with the other factors was 0.206 (indexes ranging from 0.128 to 0.317). For the second factor, this average was equal to 0.141 (from 0.083 to 0.185). The third factor correlated with the others at an average of 0.176 (from 0.140 to 0.241). The average correlation coefficient of the fourth factor with the others was equal to 0.176 (from 0.140 to 0.241). The average correlation for the fifth factor was 0.121 (from 0.032 to 0.128).

Internal consistency reliability for each scale was computed with Cronbach’s alpha and the corresponding SEM (Standard Errors of Measurement) For Fearfulness, α = 0.93 (SEM = 0.24), for Friendliness, α = 0.91 (SEM = 0.22), for Playfulness, α = 0.79 (SEM = 0.44), for Aggressiveness, α = 0.95 (SEM = 0.21) and for Opportunism, α = 0.76 (SEM = 0.30) were the alpha values. The McDonald’s omega coefficient for the Fearfulness factor was slightly higher than alpha at 0.94. For the other factors, the omega coefficients did not differ from the alpha coefficients.

### Personality traits and behavior

We found that all personality traits correlated with the observed behaviors in bonnet macaques, but the number of correlations was different for each trait (Table 2). The highest number of correlations were found for Playfulness (15 coded behaviors, including 7 affiliative, 6 aggressive, 1 communicative and 1 neutral), Opportunism (13 coded behaviors, including 5 affiliative, 3 aggressive, 4 communicative and 1 neutral) and Fearfulness (12 coded behaviors, including 6 affiliative, 3 aggressive, 2 communicative and 1 neutral). For Friendliness, relationships were found with 8 behaviors (including 3 affiliative, 3 aggressive, 2 communicative). Finally, Aggressiveness was associated with 3 aggressive and 3 communicative behaviors.

**Table 2:** Correlations matrix showing relationship between personality trait and behavior in bonnet female macaques in Thenmala, southern India (*n* = 32) ^a, b ​^.

Behaviors	Personality trait				
	Fearfulness	Friendliness	Playfulness	Aggressiveness	Opportunism
***Affiliative*** approach	− 0.441**	0.332*	0.259	0.040	0.318*
coalition support	− 0.105	0.319*	0.228	− 0.047	0.161
grab	− 0.346*	0.128	0.376*	0.286	0.470**
groom	− 0.488**	0.311*	0.286	0.104	0.477**
hug	− 0.408**	0.135	0.183	0.027	0.337*
mate	− 0.008	0.171	0.356*	0.178	0.080
mock bite	− 0.320*	0.253	0.581***	0.292	0.326*
mount	0.167	− 0.223	0.375*	0.217	0.252
muzzle contact	− 0.142	0.215	0.346*	− 0.052	0.104
play	− 0.217	− 0.016	0.554***	0.098	0.238
sleep on	− 0.470**	0.208	0.298*	0.050	0.266
***Aggressive*** aggressive approach	0.349*	0.025	− 0.021	− 0.274	− 0.022
avoid	− 0.194	0.447**	0.491**	− 0.075	0.217
chase	0.302*	− 0.198	0.454**	0.098	0.133
displacement	0.073	0.133	0.304*	− 0.146	0.165
flee	− 0.172	0.343*	0.439**	− 0.121	0.276
lunge	0.178	− 0.194	0.192	0.342*	0.047
pull	− 0.145	− 0.079	0.125	0.408**	0.343*
push	− 0.361*	0.031	0.174	0.224	0.336*
slap	− 0.225	− 0.086	0.418**	0.398*	0.405*
threat	− 0.062	0.438**	0.322*	− 0.165	0.163
***Communicative***					
contact rattle, woo	0.358*	0.056	− 0.021	0.035	− 0.306*
scream, squeak	− 0.068	0.473**	0.406*	− 0.113	0.299*
threat rattle	− 0.351*	0.030	0.291	0.385*	0.361*
open mouth threat	− 0.224	0.046	0.175	0.296*	0.324*
brow raise	0.013	− 0.328*	0.036	0.591***	0.134
***Neutral***					
self-scratch	− 0.355*	0.161	0.280	− 0.069	0.157
yawn	0.165	− 0.194	− 0.357*	− 0.224	− 0.305*

Note.^a^ Behaviors not significantly associated with any personality trait: inspection, looking at, neutral touch, alarm call, lip smack, bared teeth, teeth chatter; ^b^ Pearson correlation coefficient, one-tailed significance: * < 0.05; ** < 0.01; *** <0.001.

### Personality and sex, age and dominance rank

Results of relationships between sex, age and personality traits are presented in Table 3.

Females were significantly higher in Friendliness than males. They were also significantly lower in Aggressiveness. Younger individuals were significantly higher in Playfulness than older ones. No interaction effects were found for Friendliness, Aggressiveness, Playfulness, and Fearfulness, with the latter (Fearfulness) being also independent of both sex and age.

**Table 3:** Relationship between age, sex and personality trait in two groups of bonnet macaques in Thenmala, southern India.

Personality	Females			Males			Sex, age, interaction effect		
	**Subadult**Μ (SD) *n* = 6	**Adult** Μ(SD) *n* = 32	**Total** Μ (SD) *n* = 38	**Subadult** Μ (SD) *n* = 7	**Adult** Μ(SD) *n* = 19	**Total** Μ(SD) *n* = 25	**Sex effect** **F(1**,**59)**	**Age effect** **F(1**,**59)**	**Interaction effect** **F(1**,**59)**
Friendliness	5.44 (0.30)	4.95 (0.48)	5.03 (0.49)	4.02 (0.47)	4.30 (0.93)	4.22 (0.83)	26.99 p *<* 0.001	0.29 *p* = 0.590	3.71 *p* = 0.059
Aggressiveness	3.19 (0.66)	3.32 (0.72)	3.30 (0.70)	3.90 (0.53)	3.73 (1.27)	3.78 (1.10)	4.05 p *=* 0.049	0.01 *p* = 0.944	0.30 *p* = 0.587
Playfulness	5.86 (0.72)	4.32 (0.74)	4.56 (0.92)	5.10 (0.58)	4.44 (0.72)	4.62 (0.74)	1.98 *p* = 0.165	23.68 *p* < 0.001	3.78 *p* = 0.057
Fearfulness	2.82 (0.85)	3.02 (0.71)	2.99 (0.72)	3.38 (0.49)	3.59 (1.33)	3.53 (1.15)	3.74 *p* = 0.058	0.46 *p* = 0.498	0.001 *p* = 0.990
Opportunism	5.52 (0.24)	5.03 (0.63)	5.10 (0.61)	4.67 (0.60)	5.07 (0.65)	4.96 (0.65)	4.44 *p* = 0.039	0.06 *p* = 0.804	5.36 *p* = 0.024

For Opportunism, both sex and interaction effects were significant. Simple effects analysis (after applying Sidak’s multiple testing correction) revealed that sex was significant only among subadults, with females showing higher levels than males (*p* = 0.015). Among adults, the sex effect was not significant (*p* = 0.825). The dominance rank impact was observed in Aggressiveness only, as it differentiated high- from mid-ranking females (Table 4). High-ranking females had significantly higher intensity of Fearfulness than mid- and low-rank females. Finally, the intensity of traits such as Friendliness, Playfulness, and Opportunism were observed at similar levels in all females.

**Table 4:** Relationship between the dominance rank and personality trait in two groups of bonnet macaques in Thenmala, southern India.

Personality	High rank - Low rank		High rank- Middle rank		Middle rank- Low rank	
	*z*	*p*	*z*	*p*	z	*p*
Friendliness	0.20	0.840; (1.00)	0.29	0.769; (1.00)	0.07	0.943; (1.00)
*chi*^2^ = 0,09; *d f* = 2; *p* = 0.956						
Aggressiveness	1.86	0.063; (0.190)	2.52	0.012; (0.035)	0.48	0.634; (1.00)
*chi*^2^ = 6.80; *df* = 2; *p* = 0.033						
Playfulness	1.20	0.230; (0.690)	0,83	0.408; (1.00)	2,06	0.040; (0.119)
*chi*^2^ = 4.23; *df* = 2; *p* = 0.120						
Fearfulness	2.45	0.014; (0.043)	2.25	0.024; (0.073)	0.41	0.685; (1.00)
*chi*^2^ = 7.36; *df* = 2; *p* = 0.025						
Opportunism	0.56	0.575; (1.00)	0.38	0.701; (1.00)	0.96	0.338; (1.00)
*chi*^2^ = 0,92; *d f* = 2; *p* = 0.632						

(p in brackets) – probability in Dunn tests after introducing Bonferroni correction for multiple testing. Low rank (*N* = 7), middle rank (*N* = 10); high rank (*N* = 8).

## Discussion

The aim of the study was to examine the personality structure in free-ranging groups of bonnet macaques. We showed that in bonnet macaques, the personality structure generally aligns with that described in both wild and captive macaque species. This structure includes Fearfulness/Anxiety, Sociability/Friendliness, Playfulness (following the content analysis of questionnaire items, covering two sub-dimensions: Activity and Openness, sometimes regarded as distinct personality traits^18^), and Aggressiveness/Dominance, with the exception of Opportunism^2,23,32,44^. In Assamese (*M. assamensis*) and Barbary macaques (*M. sylvanus*), Opportunism was defined as a personality trait related to jealousy, manipulation and bullying^44–46^which suggests its occurrence during group interactions with other animals, in contrast to our observations where an Opportunistic individual set its goal by itself (e.g., stealing food). In our study, Opportunism was defined as intelligent use of opportunity for personal profit (e.g., gaining limited resources, excluding the risk of losing the gain in favor of a higher-ranking individual) accompanied by vigilance and high reactivity to the environment. Its presence may stem from adaptations that favor a low-conflict lifestyle alongside humans, in contrast to rhesus macaques which also inhabit anthropogenic environments but engage in more aggressive interactions with humans and show a reluctance to compromise^47^. However, it cannot be ruled out that Opportunism is not so much a specific trait of bonnet macaques as it is a result of adaptation to a human-impacted environment where access to food is much more limited than in the captive setting. The majority of studies were conducted in captivity, which may have influenced the lack of identification of the aforementioned personality trait, as we defined it. According to Adams et al.^44^, Friendliness appears to be a unique feature for all macaques, independently of their social style. Our study confirms that bonnet macaques (Grade 3) can be characterized by this trait as well.

Alongside the questionnaire assessment of personality, for both sexes, the behavior of females was observed. This behavior was found to correspond with all above traits of personality structure of bonnet macaque that we found. It is likely that the diversity and large number of behaviors observed in the natural environment contributed to the greater number and strength of correlations in our study compared to those conducted by researchers such as Capitanio^5^. Notably, only nine behaviors were exclusively linked to a single trait, some of which are associated with other motivational systems (e.g., sexual behaviors). Thus, as in humans, a single behavior should not be expected to be highly specific to one trait, as its analysis must consider the motivational context. Our research showed that most behaviors can express various personality traits.

For the three personality traits (Fearfulness, Playfulness, Aggressiveness), the correlations with behaviors align with the content of these traits. A high level of Fearfulness is associated with avoidance of affiliative and aggressive-confrontational interactions, both communicative and actively harmful. Interestingly, aggressive approach and chase were the only aggressive behaviors positively correlated with Fearfulness, suggesting that some highly anxious individuals may respond to threats with aggression. Consistent with the content of Aggressiveness, this trait was correlated exclusively with physical aggression (e.g., pulling, slapping) and threatening communication. Playfulness, characterized by both activity and exploration, was associated with the initiation of a wide range of affiliative and aggressive behaviors, as well as high levels of arousal (negatively correlated with yawn). While these behaviors were highly diverse - spanning affiliative, sexual, and aggressive contexts - this finding supports the adaptive function of play, as it facilitates the repeated expression of behaviors critical for survival and socialization^48^. Interestingly, Opportunism, which includes vigilance and intelligence, was linked to various affiliative behaviors, aggressive behaviors (including communicative aggression), and notably, avoidance of attracting the attention of other individuals (negatively correlated with contact rattling). This constellation of behaviors suggests a sophisticated behavioral strategy that includes maintaining social ties, adopting an assertive attitude, and strategically inhibiting communication for personal gain. Another possible explanation for the links between Opportunism and highly varied behaviors is the diverse expression of this trait, likely shaped by factors such as rank, situational context, or the intensity of other personality traits. Regarding Friendliness, which encompasses caring and warmth in social interactions, the pattern of correlations is particularly intriguing, as it is not immediately intuitive from an anthropocentric perspective. While Friendliness, as defined by its components, was associated with affiliative behaviors, it also correlated with submissive behaviors (e.g., avoidance, fleeing, screaming/squeaking) and threatening communication. This finding suggests that a friendly disposition does not preclude avoiding contact with others or issuing threats to prevent overt conflict.

We found that regardless of age, females showed higher Friendliness than males, which is a consequence that bonnet macaque females form stable and matrilineally heritable dominance ranks for whole life^38^. Therefore, their relationship with other females is based on strong foundations. Moreover, as higher levels of socialization/friendliness in females translate into having more offspring and survival^49^, we can expect that bonnet macaque females are social to maximize their reproductive success, in contrast to males, who typically disperse into groups with no relatives, and compete for the rank and access to females. However, there is a lack of research assessing fitness benefits related to personality variability^50,51^with some exceptions, such as captive male gorillas where Extraversion was linked with longer survival^52^.

For males of many primate species, being aggressive and dominant may help to gain access to limited resources, such as estrous females. However, unlike other macaque species (e.g. lion-tailed macaques *M. silenus*, or rhesus macaques)^39,53^(except for Assamese macaques, i.e^44^). , male bonnet macaques form coalitions based on multiple affiliative behaviors^39,54,55^. Our results confirmed their high tolerance level^33^as males had only slightly higher Aggression than females, and there was no change in Aggression level between subadult and adult males, in contrast to other macaque species e.g^16^.

We found, as expected, that Playfulness was age-dependent, as it was more common for subadult than adult animals. This result is not surprising, as play motivated by an exploratory drive is a universal characteristic of young individuals, both human and animal, and it is consistent with the assumptions of developmental theories about the high importance of the exploratory drive in children^56^. The exploratory drive is responsible for active interaction with the environment based on the reward system, and therefore promotes learning, performance, and cognitive mastery of the environment leading to increased adaptation to it^57^.

Another trait that characterized bonnet macaques, Fearfulness, was independent of sex and age, which may suggest that the level of this factor is highly individualized. The higher score of Fearfulness, however, was observed in low-rank individuals, which may be related to the consequences of their position in the group, as often the low-ranking individuals are the receivers of aggression in the group and may not have support from other individuals^58^.

Opportunism, defined as intelligent use of opportunity for personal profit outside group interactions, accompanied by vigilance and high reactivity to the environment, may be characteristic of bonnet macaques and be related to various social factors, such as sex, age, social rank or living in anthropogenic habitat. In our study groups, subadult females showed higher Opportunism than males, but there was no sex difference in adults. Opportunism may be more understandable in the context of access to limited resources (e.g., access to limited anthropogenic food). The position of subadult females in the group is lower than subadult males, or even the low-ranking adult females, which means that their cleverness and seizing opportunities that other individuals miss, may be promoted to steal food.

Our research leads to several important conclusions. A comparison of observed behavior with questionnaire ratings based on the judges’ cumulative experience confirmed high behavioral convergence with three traits, namely Fearfulness, Playfulness and Aggressiveness. In the case of Friendliness and Opportunism, it is important to conduct further studies to understand the relationship of these traits with the functional value of behavior and to test whether the behavioral expression of this trait is typical for the entire species or only for females. Our research demonstrated that the tolerant style of bonnet macaques is reflected both in their personality structure (Opportunism was not associated with aggression-related characteristics) and in the lack of pronounced differences in Aggression between sexes as well as between younger and older males. Our research suggests that a cooperative and socially bonded style in both sexes is as adaptive as the competitive and dominance-based behavior typical of males.

## Methods

### Study site and study subjects

We studied two habituated bonnet macaque groups (Dam and Eco) at Thenmala Dam and Ecotourism Recreational Area (8°57’37” N, 77°03’40” E) on the outskirts of Thenmala, Kerala, southern India, from January 2020 to February 2022. The home ranges of the study groups included small forest patches, three villages, governmental forest offices, and an ecotourism center. The Dam group consisted of 35 individuals and the Eco group consisted of 59 individuals (Table 5). There are several non-habituated groups of bonnet macaques in the area.

**Table 5:** Composition of bonnet macaque study groups at the beginning of the observation period in 2020, in Thenmala, southern India.

	Dam	Eco
Group size	35	59
Adult females	10–13	7–13
Adult males	1–8	6–14
Subadult females	0–2	3
Subadult males	2–5	1–2
Juveniles (both sexes)	4–8	16–21
Infant females	2–7	1–2
Infant males	1–5	5–7

In behavioral and personality ratings, our subjects were both subadult (3–4 years) and adult females (≥ 4 years) from both groups. Subadult (4–5 years) and adult (≥ 5 years) males from Dam and Eco groups, however, were rated in personality ratings only. All macaques were habituated to human presence and individually recognizable using natural markings such as facial characteristics (color, scars, wrinkles), relative body size, and nipple color and size.

Bonnet macaques exhibit pronounced reproductive seasonality, with mating and births occurring primarily during specific times of the year^38^. Seasonal reproduction has significant implications for male behavior and social dynamics within bonnet macaque groups. During the mating season, competition among males for access to fertile females intensifies. Higher-ranking, more dominant males often monopolize mating opportunities, using aggressive displays and physical confrontations to assert dominance and exclude lower-ranking rivals^38^. Males may also form strategic alliances and coalitions to improve their chances of mating success. Outside the mating season, male-male aggression tends to decrease as the focus shifts to other activities like foraging and grooming^38^.

### Behavioral observations

Teams of two or three observers at a time conducted behavioral observations of all females five days a week during a 15-min focal sampling at a distance of approximately 3–10 m from the monkeys. The observers reached inter-observer reliability in individual recognition, focal sampling, and all occurrences sampling prior to commencing data collection (range of Cohen’s Kappa: 0.91–0.98). The focal sampling order in each group was determined opportunistically while taking care to balance morning and afternoon sampling.

At any time period, members of the team observed different females and communicated with each other throughout the day about which individuals had and had not yet been followed. During the focal sampling, we recorded data on aggression (e.g., chase, bite, slap, threat), affiliation (e.g., grooming, hugging) and vocal and non-vocal communication behaviors. While collecting data, we noted the identity of the monkey the focal individual was interacting with, the time when the interaction occurred and, for grooming and hugging only, the duration of the interaction. Additionally, we collected self-scratch and yawn (neutral behaviors).

Over 23 months, we completed 3723 15-min focals (930.75 h of observations). Data was entered into Samsung Galaxy Tablets using customized data forms created in HanDBase^®^ (DDH software).

### Dominance ranks of the females

Dominance ranks were based on 750 recorded dyadic agonistic interactions (Dam: 474, Eco: 276). Agonistic interactions included non-physical threats (e.g., facial displays), approach–avoids (moving away from another who is approaching), supplants (taking the place of another), physical contacts (e.g., biting, tail-pulling, and pushing), and chases (aggressively pursuing another). From these interactions, we constructed dominance matrices for males and females in each study group following^59,60^. In the matrix, individuals in rows are ‘dominant’ and those in columns are ‘subordinate’, and an entry in a cell indicates the number of fights won by a row individual out of the total number of encounters that occurred between the row and the column individuals (for more details on methodology, see Anand et al.^61^).

### Personality ratings

After at least three to six months of behavioral data collection in the two study groups of bonnet macaques, each field assistant was asked to rate the all subadult and adult males and females. Personality ratings for each macaque were obtained from minimum two up to six field assistants. Each subject was rated on a 7-point scale for 50 personality attributes/adjectives (adapted from Stevenson-Hinde & Zunz^16^, King et al.^62^, Stevenson-Hinde et al.^63^) e.g. “Active/Energetic moves about a lot, distance traveled by walking, running, climbing, or jumping; not lethargic.” The scales used to assess personality were based on a combination of adjectives and behavioral definitions, which, according to Uher et al. 10, contributes to better prediction of behavior, compared to scales based on adjectives alone, the interpretation of which can be quite subjective.

In assessing each macaque, raters were instructed to consider its ‘usual’ behavior, relative to the other macaques in the group. One point was assigned in the absence or negligible amounts of the attribute, four points in its average manifestation, and seven points in the maximal manifestation of the attribute. All questionnaires were in English.

### Data analysis

Inter-rater consistency was measured using the intraclass correlation coefficient (ICC), which assesses reliability by comparing trait variability across all raters. A 2-way mixed effect model (3:k)^64^ was applied. Mean trait ratings for every questionnaire item for every individual among all observers was used.

Ratings on questionnaires that obtained sufficient consistency as measured by the ICC index were included in exploratory factor analysis with oblique axis rotation. Factor analysis identified five components (Fearfulness, Friendliness, Playfulness, Aggressiveness, and Opportunism). The label of each factor was derived from a content analysis of the adjectives, which had high factor loadings on a given component (at least $\:\left|0.4\right|$) and low factor loadings on the other four components (below $\:\left|0.4\right|$). Each of the five personality scales was created by summing and then averaging the values for all those adjectives that defined a particular factor. As a result, an individual’s score on each personality scale ranged from 1 to 7. The first factor, named Fearfulness, described individuals who were prone to react with anxiety, tense, and reluctant to engage in new social or non-social situations (adjectives included: nervous, tense, timid, fearful, apprehensive, insecure, submissive, solitary and cautious). Friendliness, the second factor, related to a supportive, open and warm attitude toward others and described the individuals who were caring and protective (adjectives included: nurturant, understanding, protective, warm, gentle, tolerant, sensitive, affiliative, popular, sociable and by the opposite of the adjective unemotional). The third factor - Playfulness - related to individuals who were active, initiated the play, and were ready to explore new situations (adjectives included: playful, active, curious, independent and by the inverse of the adjective slow), represented a combination of Openness and Activity. Aggressiveness, the fourth component, was related to individuals prone to irritation, together with impulsive, harmful, manipulative and jealous behavior in social relations (adjectives included: irritable, aggressive, bullying, manipulative, feisty, jealous, unpredictable, defiant, impulsive, direct, reckless, effective, persistent, stingy, excitable and bold). Opportunism, the fifth dimension, was defined as intelligent use of opportunity for personal profit (e.g., gaining limited resources, excluding the risk of losing the gain in favor of a higher-ranking individual), accompanied by vigilance and high reactivity to the environment (adjectives included: vigilant, opportunistic, intelligent and by the inverse adjectives lazy, depressed and equable). Details of the factor loading matrix are presented in Table 2.

In the subsequent stage of analysis, the association between personality traits and behaviors, exclusively obtained from female subjects, was examined. The frequencies of 35 recorded behaviors were standardized by normalizing them with respect to the duration of observation for each individual. Four macaques, observed for less than 8 h, were excluded from the analysis. For the remaining 32 individuals, correlation coefficients between the frequency of specific behaviors and five personality traits were calculated.

In the next step of the analysis, we checked whether the scores obtained by each factor (personality trait) were related to age (subadult/adult) and sex (male/female) of the individuals. For this purpose, a 2-way analysis of variance (in the version for classified variables) was used to estimate two main effects (age and sex) and the interaction effect of the independent variables used.

Adult females were then divided into three groups based on dominance rank (low, middle and high). We used the Kruskal-Wallis test and Dunn’s post hoc tests to test whether dominance rank differentiated the scores obtained on each of the five factors (personality scales). Bonferroni’s multiple testing adjustment was applied to the Dunn tests.

## Electronic supplementary material

Below is the link to the electronic supplementary material.

Supplementary Material 1

## Data Availability

The data used in this study are available from the corresponding author on reasonable request.
