# Supplementary material for: Personality traits and their correlation with behavior in the bonnet macaque (Macaca radiata) in southern India
Source: Sci Rep. 2024 Dec 28;14:31071. doi: 10.1038/s41598-024-82283-0 (PMC11681235; doi:10.1038/s41598-024-82283-0)
Supplement: Supplementary file 1 — Supplementary Material 1 [file 41598_2024_82283_MOESM1_ESM.docx]

**Appendix**

Appendix Table A1. Intraclass Correlation Coefficients (ICC). Mean of raters (3;k) and single raters (3;1) – two-way mixed effect model; Consistency.

| **Nr** | **Trait** | **ICC average measure (3;k) and (in parentheses)**  **ICC single measure (3;1)** | | | | | **KMO** |
| --- | --- | --- | --- | --- | --- | --- | --- |
|  |  | **6 raters** | **5 raters** | **4 raters** | **3 raters** | **2 raters** |  |
| **1** | Active | .94 (.73) | .95 (.78) | .92 (.75) | .89 (.72) | .91 (.83) | .688 |
| **2** | Affiliative | .91 (.64) | .92 (.69) | .87 (.64) | .83 (.62) | .88 (.76) | .566 |
| **3** | Aggressive | .94 (.74) | .93 (.72) | .89 (.67) | .85 (.65) | .91 (.83) | .737 |
| **4** | Apprehensive | .81 (.42) | .88 (.61) | .86 (.61) | .88 (.71) | .91 (.64) | .461 |
| **5** | Bold | .94 (.72) | .94 (.75) | .90 (.68) | .88 (.71) | .91 (.84) | .796 |
| **6** | Bullying | .96 (.82) | .96 (.81) | .92 (.75) | .91 (.76) | .92 (.85) | .582 |
| **7** | Calm | .89 (.57) | .91 (.67) | .85 (.58) | .78 (.54) | .83 (.71) | .660 |
| **8** | Cautious | .83 (.45) | .87 (.57) | .76 (.44) | .69 (.43) | .81 (.68) | .706 |
| **9** | Confident | .93 (.69) | .93 (.74) | .89 (.66) | .89 (.74) | .90 (.81) | .672 |
| **10** | Curious | .64 (.23) | .82 (.48) | .73 (.41) | .74 (.49) | .78 (.64) | .763 |
| **11** | Defiant | .82 (.66) | .89 (.63) | .83 (.55) | .71 (.45) | .75 (.60) | .605 |
| **12** | Depressed | .93 (.69) | .90 (.65) | .84 (.57) | .81 (.57) | .89 (.79) | .614 |
| **13** | Direct | .96 (.80) | .96 (.81) | .94 (.80) | .92 (.79) | .95 (.91) | .894 |
| **14** | Effective | .87 (.82) | .96 (.82) | .94 (.79) | .91 (.77) | .92 (.85) | .790 |
| **15** | Equable | .86 (.51) | .86 (.55) | .80 (.50) | .75 (.51) | .86 (.76) | .473 |
| **16** | Excitable | .83 (.45) | .85 (.53) | .80 (.50) | .75 (.50) | .74 (.59) | .614 |
| **17** | Fearful | .92 (.65) | .94 (.74) | .90 (.70) | .89 (.73) | .91 (.84) | .630 |
| **18** | Feisty | .93 (.70) | .92 (.69) | .88 (.66) | .84 (.63) | .90 (.81) | .826 |
| **19** | Gentle | .89 (.58) | .88 (.59) | .83 (.54) | .79 (.56) | .86 (.75) | .487 |
| **20** | Impulsive | .96 (.78) | .95 (.79) | .93 (.76) | .90 (.76) | .95 (.90) | .649 |
| **21** | Independent | .86 (.50) | .84 (.51) | .73 (.40) | .62 (.35) | .78 (.64) | .587 |
| **22** | Insecure | .81 (.42) | .85 (.54) | .77 (.46) | .73 (.48) | .74 (.58) | .713 |
| **23** | Intelligent | .83 (.44) | .82 (.47) | .78 (.48) | .76 (.52) | .73 (.57) | .474 |
| **24** | Irritable | .94 (.72) | .93 (.73) | .90 (.69) | .88 (.70) | .93 (.87) | .670 |
| **25** | Jealous | .93 (.70) | .91 (.67) | .86 (.60) | .82 (.50) | .89 (.79) | .534 |
| **26** | Lazy | .96 (.79) | .94 (.77) | .91 (.71) | .89 (.74) | .91 (.84) | .462 |
| **27** | Manipulative | .89 (.57) | .87 (.58) | .80 (.50) | .76 (.51) | .87 (.76) | .677 |
| **28** | Nervous | .88 (.55) | .94 (.75) | .89 (.67) | .87 (.70) | .90 (.81) | .719 |
| **29** | Nurturant | .86 (.50) | .88 (.60) | .83 (.54) | .80 (.58) | .87 (.77) | .495 |
| **30** | Opportunistic | .89 (.59) | .87 (.56) | .82 (.54) | .78 (.54) | .85 (.73) | .760 |
| **31** | Persistent | .91 (.64) | .92 (.70) | .87 (.62) | .78 (.55) | .83 (.70) | .550 |
| **32** | Playful | .87 (.52) | .94 (.77) | .93 (.78) | .92 (.80) | .94 (.88) | .521 |
| **33** | Popular | .81 (.57) | .92 (.69) | .88 (.65) | .85 (.65) | .87 (.77) | .777 |
| **34** | Protective | .86 (.51) | .87 (.57) | .78 (.48) | .73 (.47) | .85 (.75) | .766 |
| **35** | Reckless | .95 (.74) | .94 (.75) | .92 (.74) | .92 (.76) | .94 (.89) | .638 |
| **36** | Sensitive | .87 (.53) | .88 (.59) | .85 (.59) | .78 (.54) | .78 (.65) | .461 |
| **37** | Slow | .88 (.56) | .88 (.60) | .83 (.55) | .79 (.55) | .86 (.76) | .441 |
| **38** | Sociable | .90 (.60) | .90 (.64) | .88 (.64) | .85 (.66) | .92 (.85) | .591 |
| **39** | Solitary | .95 (.75) | .95 (.79) | .90 (.70) | .89 (.73) | .90 (.81) | .600 |
| **40** | Stingy | .92 (.65) | .91 (.67) | .89 (.66) | .79 (.55) | .86 (.76) | .500 |
| **41** | Strong | .96 (.78) | .96 (.83) | .93 (.78) | .92 (.79) | .90 (.82) | .676 |
| **42** | Submissive | .93 (.68) | .93 (.73) | .90 (.69) | .89 (.74) | .91 (.83) | .862 |
| **43** | Tense | .90 (.59) | .91 (.67) | .83 (.56) | .85 (.65) | .84 (.73) | .766 |
| **44** | Timid | .90 (.59) | .90 (.65) | .85 (.59) | .84 (.64) | .85 (.74) | .780 |
| **45** | Tolerant | .87 (.54) | .85 (.53) | .77 (.46) | .68 (.41) | .83 (.70) | .673 |
| **46** | Understanding | .81 (.41) | .72 (.34) | .73 (.40) | .71 (.45) | .70 (.54) | .462 |
| **47** | Unemotional | .88 (.55) | .88 (.60) | .85 (.59) | .81 (.59) | .82 (.70) | .568 |
| **48** | Unpredictable | .94 (.72) | .93 (.74) | .90 (.70) | .88 (.71) | .91 (.84) | .686 |
| **49** | Vigilant | .78 (.37) | .81 (.47) | .71 (.38) | .75 (.50) | .75 (.60) | .521 |
| **50** | Warm | .92 (.67) | .93 (.72) | .89 (.66) | .87 (.69) | .90 (.82) | .619 |
| **N (observed monkeys)** | | 29 | 33 | 41 | 52 | 62 |  |

None of the 95% Confidence Interval for ICC (3;k) and ICC (3:1) population values contain zero. All KMO indexes are > 0.4;

Appendix Table A2. Correlations between extracted factors after oblimin rotation.

|  | **Fearfulness** | **Friendliness** | **Playfulness** | **Aggressiveness** |
| --- | --- | --- | --- | --- |
| **Friendliness** | -.317 |  |  |  |
| **Playfulness** | .136 | -.083 |  |  |
| **Aggressiveness** | .241 | .161 | .161 |  |
| **Opportunism** | .128 | .032 | .185 | .140 |
